# The Magic Pudding: Comment on "Four Challenges That Global Health Networks Face"

**DOI:** 10.15171/ijhpm.2017.76

**Published:** 2017-07-05

**Authors:** Jill White

**Affiliations:** Faculty of Nursing and Midwifery, University of Sydney, Sydney, NSW, Australia.

**Keywords:** Networks, Health Policy, Policy Analysis, Politics, Power

## Abstract

This commentary reflects on the contribution of this editorial and its "Three Challenges That Global Health Networks Face" to the totality of the framework developed over the past decade by Shiffman and his collaborators. It reviews the earlier works to demonstrate that the whole is greater than the sum of the parts in providing a package of tools for analysis of network effectiveness.Additionally the assertion is made that the framework can be utilised in reverse to form a map for action planning for network activity around a potential health policy issue.

There is richness in this article^1^ in its own right. It provides a synthesis of the analysis of the multiple paired cases and shines light on four key aspects of decision-making/taking by networks. That in itself is an important contribution to which I will return later. For me the added richness this editorial brings is in the development of yet another element to the simple picture of a complex reality Shiffman has constructed over the past decade. It provides us with intellectual tools for analysing and indeed establishing networks for global action. The elegance of Shiffman’s frameworks lies in the sophisticated simplicity, but simplicity lends itself at times to only superficial appreciation, hence the felt need for this expository commentary.

## The Roots of “Four Challenges”

Returning to the roots of Shiffman’s writings on social constructionist framing of network effectiveness enables us to appreciate this latest work, not as a standalone analysis of the challenges facing global networks, but to appreciate this addition in terms of its having grown logically from the solid roots of a larger body of work. I first fell upon Shiffman’s^2^ 2007 four concept framework (actor strength, power of ideas used to portray the issue, issues characteristics themselves, and the political context), when seeking to understand the lack of success of a large health professional group in having influence in health policy. This may seem a long way away from the framework’s intended use, nevertheless, it provided, even in its early form, a window into the socio-politics of policy making and network effectiveness. The framework was embellished in the 2009^3^ work with emphasis on the importance of the portrayal of the ideas by the actors/ network, of their framing of the issue. It demonstrated the power of the social construction of issue framing, and the power and prominence of this, as opposed to the traditional understanding of the importance of rational/objective factors, such as burden of disease. In 2015, Shiffman et al^4^ presented the comprehensive ten factor framework for analysis of global health network effectiveness which further developed the earlier thinking, expanding on the actor/network features (leadership, governance, composition and framing strategies); the policy context (allies and opponents, funding availability and global expectations of the issue’s priority); and thirdly the issues characteristics themselves (severity, tractability and the groups affected). Again I leapt on the work seeing not only its direct relevance to understanding a complex global health landscape but also its applicability to further illuminate the positioning of the Nursing profession in relation to policy influence. For decades nurses had written about the need for greater input into policy^5-10^ and for years others, in positions of global health influence themselves, outside the discipline had called for greater nursing involvement.^11-17^ The Shiffman framing4 enabled examination of the internal issues for the profession, including leadership, framing, and power. It highlighted Nursing’s insularity and lack of coalition building, its scarce resources and at times profound political naivety.

This IJHPM editorial1 “Four challenges...” provides a capstone to Shiffman’s body of work by highlighting the evidence-based distillation of the most salient features of the 2015 model which are amenable to network/actor action to enhance effectiveness. These four challenges are related to two forms of framing: internal problem definition and its coherence and cohesiveness; and the framing of messaging for external groups portrayed to inspire action and resource commitment; thirdly, the forging of coalitions which are broad based and stable; and finally determining a cohesive governance model suited to the group and the issue. The diagrammatic representation, not unlike that of 2015 work, does not, however, capture the dynamism and interactive nature of the framework elements, hence I present this in an alternate depiction (Figure ). The Shiffman work in totality provides a wonderful case example of the incremental development of quite sophisticated thinking based in analysis of real world examples from global health practice and enables the latest four challenges to be seen in the context of the larger whole. This contextualising also enables the latter part of the editorial to be identified as a distillation of the responses to Shiffman’s^16,17^ earlier work on power relations and their legitimacy which, without this contextualising, appears as a somewhat abrupt afterthought in this editorial.

**Figure:**
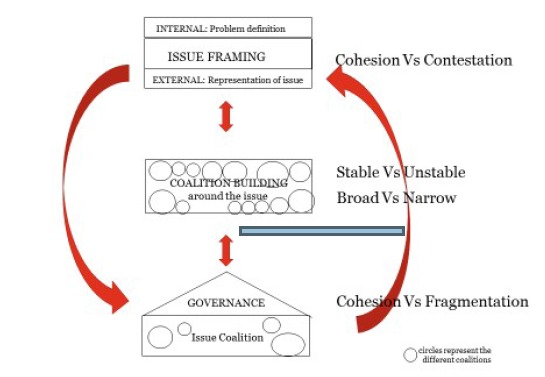
Four Challenges/ Decisions.

In this latter section on network legitimacy Shiffman1 uses the not uncommon North-South framing, usually employed by Western European or North American writers, one which I, as an Antipodean, seek to challenge. This binary essentialises the North as developed and the South as less so. Contemporary geopolitics are re-writing this old thinking, taking heed of the major advances within some African countries, acknowledging the Southern existence of Australia and New Zealand and challenging the North’s position in view of Eastern European changes, North Korea and bizarre reversals of movement to universal health coverage in the United States. This binary also ignores the commonalities in equatorial areas, North and South, in which so many contemporary health related issues are appearing, such as displaced persons and climate effects.

## New Applications of the Framework

The work of Shiffman,^1,4^ I believe, can also be utilised as a process for the development of plans for network formation and action on health issues. When used in reverse order the three major components of the 2015 framework enables actors to determine directions for decision making. Beginning with the issue: its tractability, severity and the identification of the affected groups, a primary decision can be made – is the issue potentially amenable to action, and if so given the “problem claim” what is the “solution claim”? If these responses suggest a way forward then the question becomes: Is the environment conducive at the present time? Who are the potential allies and opponents – a traditional stakeholder analysis^18^? Is there funding potentially accessible and would the external environment see that this network/group as having a legitimate role in addressing the issue? Lastly, and very importantly, is the need to address the characteristics of the internal actor/network environment. This suggest the need to answer the following questions: does the group have the necessary leadership? Is the composition of the group coherent and cohesive in their internal problem framing of the issue? Who, outside usual health related suspects, should be involved in any coalition which could be built? Is there an existing coalition with whom to join and if so what would the unique contribution be? How can the coalition be formed which will provide breadth and stability? Finally, how can the issue be framed such that it will gain traction with the various audiences who need to be persuaded to action, whether related to funding, other resources or smoothing the path towards policy action and success?

This reverse framework process, it is planned, will be trialled at an upcoming policy summit which will explore the question “what should nursing’s role be in our current humanitarian and human rights issues?” Hopefully the outcomes will be the subject of a later conversation in IJHPM.

There is a much loved Australian children’s book from the time of the depression which has as a main character a magic pudding.^19^ The pudding walks the country backroads meeting up with “swaggies” – men seeking food and shelter and work, and of course in any good children’s book they are some “baddies.” The pudding is eaten by the men but with each slice taken the pudding magically becomes whole again. It is known as the “cut and come again pudding.” Shiffman’s work reminds me of “The Magic Pudding.” With each new element in thinking and analysis it provides another richer set of tools for us to use in striving to improve outcomes in some of our major global health issues. It also helps groups currently not engaged as productively as they should be determine what they need to do exert appropriate influence. In these respects, like the magic pudding, it is the gift that keeps on giving.

## Ethical issues

Not applicable.

## Competing interests

Author declares that she has no competing interests.

## Author’s contribution

JW is the single author of the paper.
